# Climate change and 2030 cooling demand in Ahmedabad, India: opportunities for expansion of renewable energy and cool roofs

**DOI:** 10.1007/s11027-022-10019-4

**Published:** 2022-08-08

**Authors:** Jaykumar Joshi, Akhilesh Magal, Vijay S. Limaye, Prima Madan, Anjali Jaiswal, Dileep Mavalankar, Kim Knowlton

**Affiliations:** 1 Gujarat Energy Research and Management Institute (Former), Gandhinagar, India; 2 Natural Resources Defense Council, New York, NY USA; 3 Indian Institute of Public Health, Gandhinagar, Gandhinagar India; 4 Mailman School of Public Health, Columbia University, New York, NY USA

**Keywords:** India, Climate change, Adaptation, Cool roofs, Air conditioning, Energy demand, Electricity, Renewable energy

## Abstract

Most of India’s current electricity demand is met by combustion of fossil fuels, particularly coal. But the country has embarked on a major expansion of renewable energy and aims for half of its electricity needs to be met by renewable sources by 2030. As climate change-driven temperature increases continue to threaten India’s population and drive increased demand for air conditioning, there is a need to estimate the local benefits of policies that increase renewable energy capacity and reduce cooling demand in buildings. We investigate the impacts of climate change-driven temperature increases, along with population and economic growth, on demand for electricity to cool buildings in the Indian city of Ahmedabad between 2018 and 2030. We estimate the share of energy demand met by coal-fired power plants versus renewable energy in 2030, and the cooling energy demand effects of expanded cool roof adaptation in the city. We find renewable energy capacity could increase from meeting 9% of cooling energy demand in 2018 to 45% in 2030. Our modeling indicates a near doubling in total electricity supply and a nearly threefold growth in cooling demand by 2030. Expansion of cool roofs to 20% of total roof area (associated with a 0.21 TWh reduction in cooling demand between 2018 and 2030) could more than offset the city’s climate change-driven 2030 increase in cooling demand (0.17 TWh/year). This study establishes a framework for linking climate, land cover, and energy models to help policymakers better prepare for growing cooling energy demand under a changing climate.

## Introduction

In India, average countrywide temperatures increased by 0.45 °C between 1986 and 2015 and by mid-century are projected to increase by 1.39 °C compared to 1976–2005 (Sanjay 2020). The frequency of high-temperature extremes has also increased between 1951 and 2015, with accelerated warming trends over recent decades (Sanjay 2020). People across India already face life-threatening heat exposures and it is estimated that moderately and extremely hot temperatures caused nearly 90,000 premature deaths in 2015 (Fu et al. 2018).

Keeping people in India cool and reducing heat-related health problems is an urgent need (Woetzel et al. 2020). Extreme heat exposure impairs cognition and is linked to poor student performance, low birthweight, and other long-term developmental issues (Xu et al. 2014). Research indicates that by mid-century, the country could suffer economic losses from heat-related problems (Woetzel et al. 2020). Air conditioning (A/C), while an important climate adaptation in India, remains accessible to only about 7–9% of housing units (Ministry of Environment, Forest and Climate Change (Government of India) 2019). Buildings in India constitute 33% of total electricity consumption (Ministry of Power 2021), and it is expected India’s total electricity demand will rise from 372 to 4697 TWh/year and buildings will demand 55% of total electricity by 2047 (Ministry of Power 2021).

The India Cooling Action Plan (ICAP) projects nationwide cooling demand to grow eightfold by 2037–2038 compared to 2017–2018. Importantly, ICAP projections are based on population and economic growth but do not consider the ways in which climate change-driven temperature increases will further stimulate increased demand for cooling in buildings. Cooling buildings using A/C saves lives and reduces heat-related illness (Romanello et al. 2021), but if the electricity to power cooling is supplied by fossil fuels, this climate adaptation response can worsen ambient air pollution (Abel et al. 2018). Currently, the energy needed to power A/C in India’s buildings is largely provided by fossil fuels, particularly coal (U.S. Energy Information Administration 2020).

Prior modeling of climate and cooling demand is dominated by global- and country-level assessments (Khosla et al. 2020; Mastrucci et al. 2021). However, there is a growing body of work on analyzing cooling energy demand projections at the city level to evaluate changing dynamics of cooling needs and designing city-specific solutions to meet the demand (Frayssinet et al. 2018; Ortiz et al. 2018; Khosla et al. 2021; Kumari et al. 2021). Studying the changing dynamics of cooling energy consumption, Khosla et al. (2021) analyzed the household-level residential cooling consumption for the city of Delhi in India and recommend policy solutions for a low carbon cooling trajectory for the city. There is a need to further explore the situation, especially in low- and middle-income countries (LMICs) expected to dominate future growth in demand for A/C (International Energy Agency 2018). Even as climate change threatens to increase energy demand for cooling in many LMICs, expansion of landcover-based cooling strategies could reduce energy-intensive A/C use.

Landcover adaptation strategies including cool roofs can moderate the effects of climate change on cooling energy demand. The term “cool roofs” applies to a broad class of technologies that function to increase surface albedo (reflectance) of buildings to deflect a higher fraction of incoming solar radiation (Kolokotroni et al. 2018). Because of their relatively low cost and flexible application of reflecting materials (e.g., solar reflective paint or mosaic tiles), cool roofs are potential low-tech solutions to help keep indoor temperatures cooler and reduce cooling demand (Akbari et al. 2011; Xu et al. 2012; Li et al. 2014; Vellingiri et al. 2020).

Many cities around the world are already deploying cool roofs, and researchers have identified a number of associated health, economic, and environmental benefits (Bhatia et al. 2011; Xu et al. 2012). Despite the potential for passive cooling technologies to serve as a cost-effective climate change adaptation strategy, there has been little evaluation of their effectiveness for reducing energy demand in buildings and their potential to help address intersecting climate and energy problems.

This investigation aims to provide city-level estimates of the cooling energy demand reductions achievable through cool roof interventions in the context of climate change-driven warming by the year 2030. We focus on Ahmedabad, a city in the Indian state of Gujarat that has pioneered actions to respond to climate-sensitive health threats (Knowlton et al. 2014). First, we analyze the opportunity to limit electricity-related power plant emissions in the region through significantly increased use of renewable energy in place of coal combustion, in alignment with India’s national climate goals (United Nations Framework Convention on Climate Change 2021). Then, we explore the potential to reduce the energy needed for cooling through expanded future deployment of cool roofs in the city.

## Methods

The aims of our integrated modeling effort are threefold: (1) to estimate the annual electricity demand of Ahmedabad by 2030, (2) to estimate the fraction of this 2030 electricity demand that would come from cooling demand in buildings, and (3) to assess the sensitivity of the modeled cooling demand to a set of future scenarios: climate change (the impact of rising temperatures due to climate change by 2030), mitigation (limiting the use of fossil fuels to meet additional cooling demand), and adaptation (implementing cool roofs at three levels of spatial coverage to assess impacts on 2030 cooling demand).

**Fig. 1:**
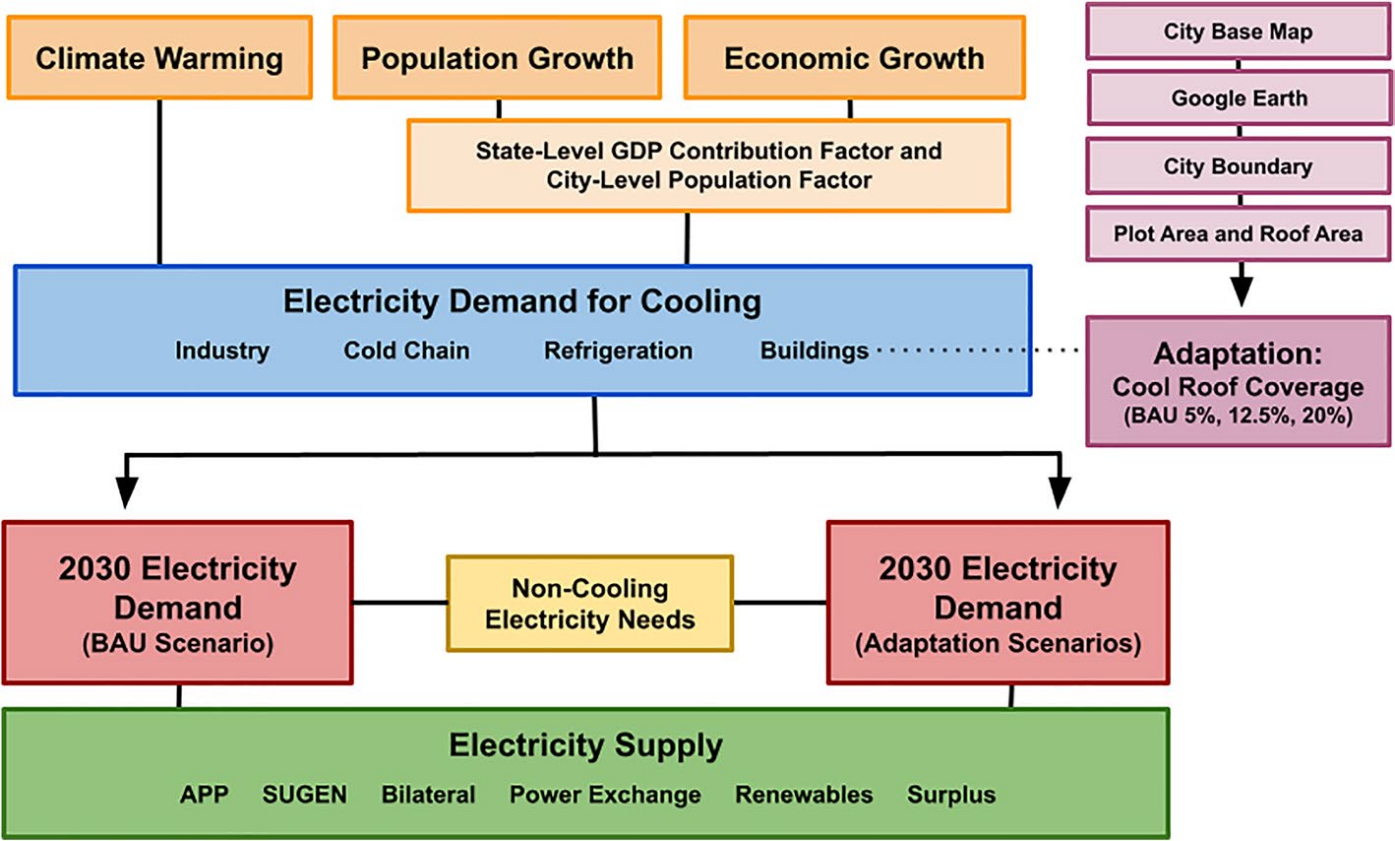
Framework for an integrated climate, cooling demand, and total energy demand emission model. We consider various levels of climate change adaptation through cool roof implementation. Abbreviations used: GDP Gross Domestic Product, BAU business as usual, APP Ahmedabad Power Plant (thermal coal), SUGEN Surat Generating Plant (gas-powered)

Our modeling was conducted through a dynamic Excel spreadsheet (see Supplemental Information). The structure of the model is displayed in Fig. 1.

### Data sources

Annual energy statistics for the state of Gujarat, and in particular, the role of distribution companies (discoms), were derived from the annual tariff orders from Gujarat Electricity Regulatory Commission (GERC) for 2015–2019 (Gujarat Electricity Regulatory Commission (Gandhinagar) (2021)) and extrapolated to 2030. We also considered the effect of bilateral transactions (i.e., agreements to exchange energy between specified sellers and buyers directly or through a trading licensee for a fixed or varying quantum of power over any time period). We also used these sources to project the future increase in overall electricity demand by extrapolating from historical data. Data for cooling demand were applied from a prior study (Kumar et al. 2018), which reports 2017 metrics and extrapolates them to 2027 country-level forecasts across four categories (space cooling, refrigeration, cold chain, industrial process cooling) under a business-as-usual scenario and an alternate “improved” scenario.

### Scope and scenarios modeled

Our study employed several assumptions to facilitate data analysis. First, because India follows a financial accounting system from April to March, traditional calendar years reported in this study refer to financial years. Therefore, 2030 results correspond to the period spanning April 2029 through March 2030. Second, mobile cooling needs were not included in our analysis because the primary fuel source is petrol or diesel as opposed to grid electricity. Model estimates in Section 2.6, 2.7, and 2.8 for the years from 2027 to 2030 were linearly projected based on historical trends.

To obtain the metrics for Gujarat and Ahmedabad, we scaled the national cooling demand estimate to a local one in the absence of state- and city-level cooling data. We employed a Gross Domestic Product (GDP) model due to the inherent energy intensity correlation to economic output (Ürge-Vorsatz et al. 2015). This simplification allowed us to estimate state-level cooling needs in 2030. However, city-level GDP data for 2030 were not available, so we employed a population-based scaling approach to estimate city effects from state-level estimates. Third, the energy distribution area of the Torrent Power Limited (TPL) Ahmedabad power plant includes certain areas of the adjoining city of Gandhinagar. It is challenging to disaggregate the precise electricity demand of Ahmedabad using public data. Our analysis therefore included the energy demand from Gandhinagar as part of Ahmedabad city demand.

Two main factors are likely to affect these 2030 cooling demand estimates. They are (1) the impact of rising temperatures due to climate change (see Section 2.7) and (2) the moderating effects of implementing cool roofs on cooling demand. In regards to cool roofs, two 2030 futures are modeled: one scenario with 12.5% of total roof area covered by cool roofs, and another with 20% cool roof coverage (see Sections 2.6 and 2.8).

### Study area

**Fig. 2:**
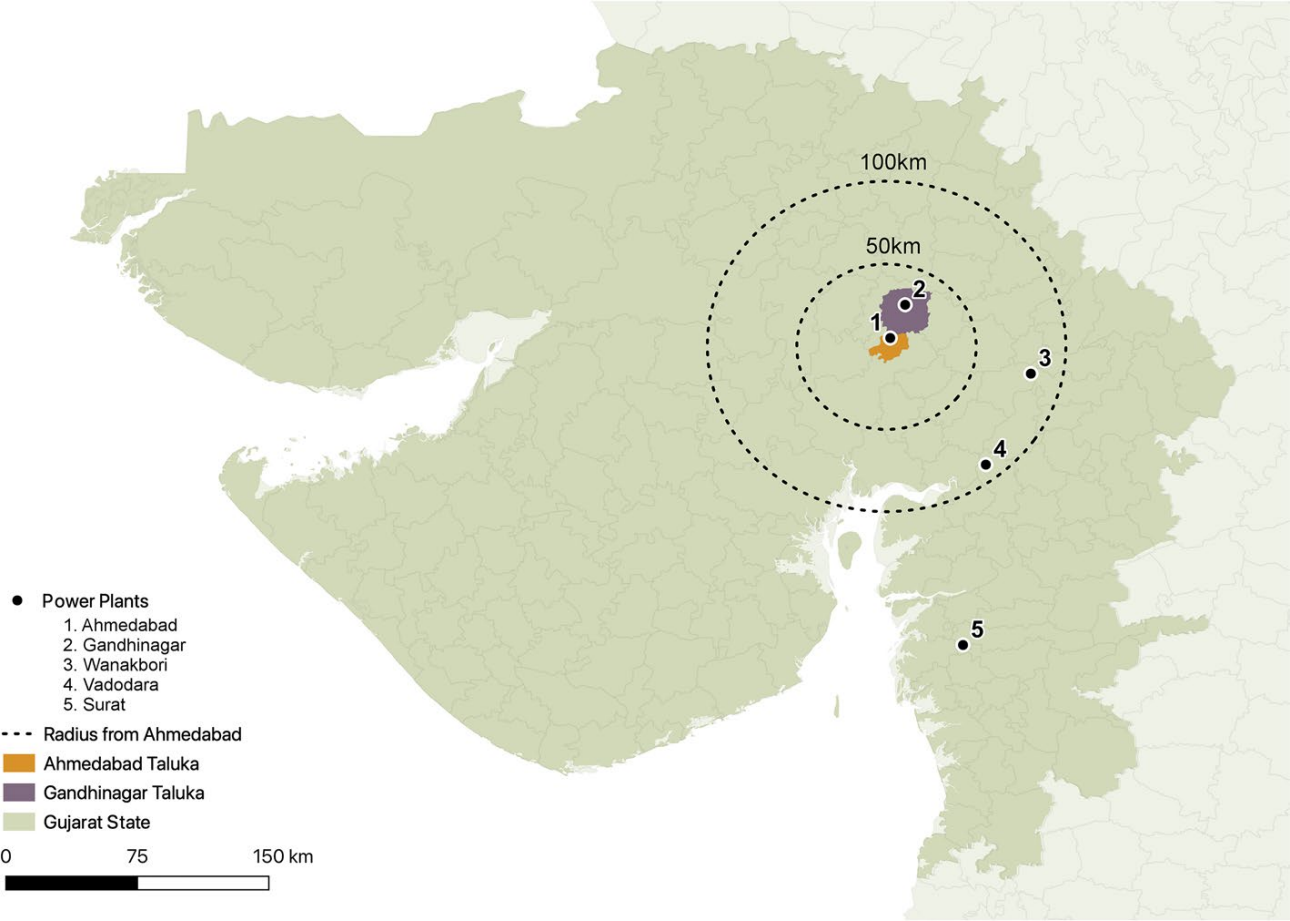
Map depicting power plants in and around Ahmedabad

For the purposes of our cool roof analysis, we defined the boundary of Ahmedabad to be the Ahmedabad Municipal Corporation (AMC) according to the city’s Draft Development Plan (Ahmedabad Urban Development Authority 2021). Electricity demand in the study area is provided by the local power distribution company (discom), TPL. The company procures energy from three principal thermal power plants: one coal-fired power plant (AMGEN) located within the AMC boundary and two natural gas power plants (SUGEN and UNOSUGEN) co-located in Surat, approximately 300 km away (see Fig. 2). The remaining electricity demand is supplied by renewable energy sources and the power exchange (see Supplemental Table S1) (Gujarat Electricity Regulatory Commission (Gandhinagar) (2021)).

Prior research in India has assumed that power plants typically impact air quality over a radius of 60–100 km, depending on prevailing winds and meteorology (Guttikunda and Jawahar 2014). Under these assumptions, two additional power plants could potentially impact the air quality of Ahmedabad. Supplemental Table S2 shows the complete list of power plants considered in our analysis. Among the six power plants that are considered in our analysis, only three supply energy (partially) to Ahmedabad. These plants are AMGEN, SUGEN, and UNOSUGEN. The remaining two plants (Gandhinagar and Wanakbori) do not supply energy to the city.

### Electricity supply and demand analysis

In 2019, India committed to installing 175 GW of renewable energy by 2022, out of which Gujarat’s share was determined at 17.1 GW total renewable energy capacity (Ministry of New and Renewable Energy, Government of India). India upgraded its national target at the UNFCCC Conference of the Parties 25th meeting in 2019 to 450 GW total installed capacity by the year 2030 (Ministry of Environment Forest and Climate Change (Government of India) (2019)). In 2021, this target was revised to 500 GW at the 26th Conference of Parties (United Nations Framework Convention on Climate Change 2021). To date, there has been no official state-wise redistribution of this updated target. To estimate the renewable energy supply by 2030, we applied the same 10% distribution of the original 175 GW target to arrive at the 45 GW Gujarat share for the revised 450 GW target by 2030 (see Supplemental Table S3).

While state targets do exist, city-level renewable energy targets are not officially determined. One of the reasons is due to the common pool of energy procurement for discoms in the state (see Section 2.1). To determine the state’s consumption of renewable energy from the overall nation’s target, we assumed the same fraction of energy demand as of 2020 to be valid in 2030. A certain portion of the energy produced from these renewable installations is allocated to Ahmedabad based on its overall energy demand. Supplemental Table S4 lists estimates of the renewable energy demand (or allocation) to Ahmedabad based on 2020 data and projected until 2030.

To estimate future electricity demand, a 2018 baseline determined from historical data was projected to 2030 by extrapolating historical growth rates. Supplemental Table S5 displays the demand growth across all consumer categories. Similarly, to project the energy supply from various sources, historical data were compiled to obtain the CAGR (compound annual growth rate), see Supplemental Table S5.

### Cooling demand analysis

Energy demand to cool buildings in India is growing faster than any other building-related energy use driver (Khosla et al. 2021). Four different categories of cooling, i.e., space cooling, refrigeration, cold chain, and industrial process cooling, were considered in this analysis, in line with a prior analysis (Kumar et al. 2018).

To calculate the total electricity consumed by cooling sectors of Ahmedabad, a combined population and GDP-based method was adopted. The Kumar et al. (2018) study only contains demand data to 2027. The demand from 2027 to 2030 was then estimated using a linear growth projection based on the CAGR in 2027. India’s sector-wise cooling demand is listed in Supplemental Table S6.

To estimate Gujarat state’s future cooling demand, we calculated a contribution factor (ratio of Gujarat’s average GDP contribution to India’s GDP from 2011 to 2019) in Supplemental Table S7. This factor is based on an assumption that economic output and energy demand are strongly correlated (Ministry of Environment, Forest and Climate Change (Government of India) 2019). The averaging approach yielded a 2030 contribution factor of 5.7% compared to 16.2% if the 2011–2019 trend is linearly extrapolated to 2030; we deploy the more conservative estimate. Multiplying India’s cooling demand with the GDP contribution factor provided sector-wise cooling demand estimates in Gujarat for 2018 and 2030 (Supplemental Tables S7 and S8). To estimate Ahmedabad’s sector-wise cooling demand from Gujarat demand data, an annual population factor was calculated, based on the ratio of Ahmedabad to Gujarat’s population (Supplemental Table S9). Each year’s population factor was then multiplied by the Gujarat annual cooling demand (Supplemental Table S8) to estimate the cooling demand in Ahmedabad (Supplemental Table S10).

### City roof area estimation

**Fig. 3:**
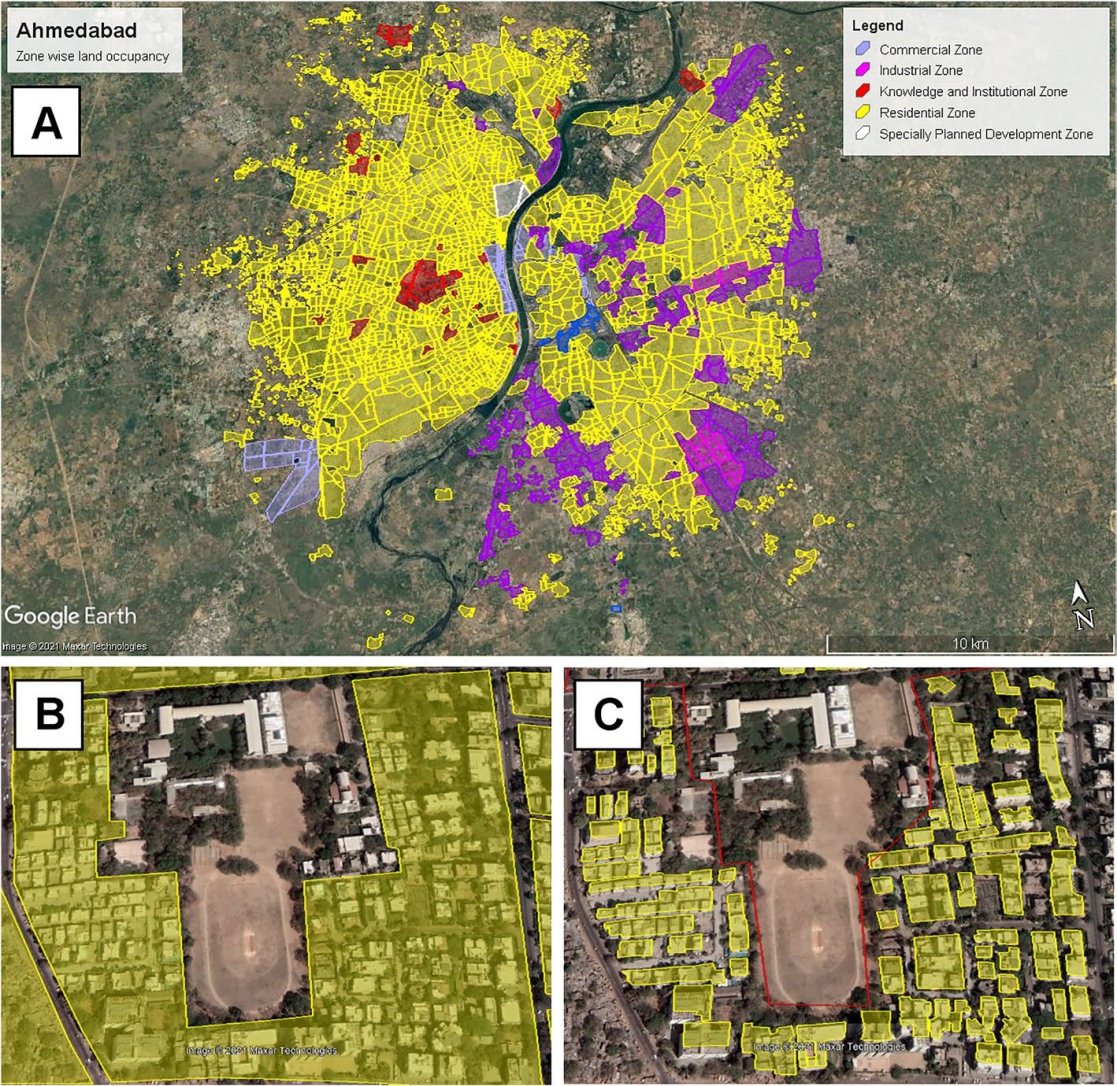
Zone-wise area under the AMC Boundary using Google Earth imagery (**A**) and method for calculating roof area from plot area using a random sampling method (**B**, **C**) (source: author’s analysis, (Google))

To determine the impact of expanded cool roof implementation on municipal energy demand, an estimate for the total roof area of Ahmedabad was required. We used a Geographic Information Systems (GIS)-based methodology to arrive at this, using publicly available data on land cover from Google Earth imagery (see Fig. 3 and Supplemental Fig. S1). The reference for plotting the areas was the development map from the Ahmedabad Urban Development Authority. This map was overlaid on the Google Earth image at the same resolution. This enabled plotting the boundary of the city of Ahmedabad.

The overlay of the development map enabled the categorization of the areas within Ahmedabad according to five main zone types: residential, industrial, commercial, knowledge and institutional, and special zones. The city zones were further divided into plots of land that have distinct ownership or boundaries. Roads and other free areas were excluded from the plots. To arrive at the roof area from the plot areas, random sampling was performed (see Fig. 3, bottom panel). Four samples per zone were taken to arrive at the ratio between the roof area to the selected plot area. To arrive at the total roof area in Ahmedabad, the baseline numbers were projected category-wise until 2030 using assumed growth rates based on the development of the city (Supplemental Table S7).

### Estimating increased cooling demand driven by climate warming

Our climate change scenario and future temperature estimate for Ahmedabad are based on National Center for Atmospheric Research data from the Global Bias-Corrected Community Earth System Model that participated in phase 5 of the Coupled Model Intercomparison Experiment (Monaghan et al. 2014). The dataset we deployed assumed Representative Concentration Pathway (RCP 8.5), which indicates a change in the annual average temperature in Ahmedabad from 300.73 K in 2018 to 301.54 K in 2030 (an increase of 0.81 °C) (Monaghan et al. 2014). While increasing deployment of renewable energy makes it somewhat less likely that future global emissions will correspond to RCP 8.5, current emission trends in developing countries still track a high emissions trajectory (Pedersen et al. 2020). For example, the International Energy Agency notes that worldwide, almost all regions registered increased carbon dioxide emissions in 2021 relative to the prior year, with the annual change exceeding 10% in India (International Energy Agency 2022). To relate modeled warming in Ahmedabad to cooling energy demand, we used the temperature vs. state-level increase in total energy metric estimated in a prior study (Harish et al. 2020) and approximated the fraction for cooling energy (Supplemental Tables S10 and S12). Our supplemental spreadsheet analysis file allows for estimation of energy demand impacts of 2 °C warming by 2030.

### Adaptation: cool roofs and energy demand

To estimate the effects of cool roof installations on cooling demand in Ahmedabad buildings, we searched the literature for estimates in other settings and calculated comparable metrics (e.g., cool roof impacts represented in annual kWh saved per square meter of cool roof area coverage). Supplemental Table S11 provides a list of these studies, spanning India, several European countries, and the United States. As Table S11 indicates, studies on this topic have identified varying energy savings impacts from cool roofs. Given the variability of these estimates, we applied the published estimate for Ahmedabad by Bhatia et al. (2011). That estimate of 14.2 kWh saved annually per square meter of cool roof coverage is comparable in magnitude to similar studies in other Indian cities including Hyderabad (Xu et al. 2012) and Delhi (Garg et al. 2016).

For this study, we estimated that a total 5% of the roof area has already been covered by some kind of reflective coating through pilot demonstration projects (Natural Resources Defense Council 2019). We deployed the cool roof energy savings metric in our analyses of future cool roof impacts on energy demand in two 2030 scenarios relative to BAU case with no additional cool roof deployment beyond current coverage: (1) a scenario in which cool roofs reach 12.5% areal coverage in the city by 2030 (a 7.5% increase from 2018 coverage) and (2) a scenario in which cool roofs expand from 5% existing areal coverage to 20% total coverage.

## Results

Our integrated modeling approach allows us to estimate the combined 2030 effects of climate warming, population growth, economic expansion, and cool roof installations. Table 1 displays estimates of historical and projected energy supply for the city based on current trends. In 2018, renewable energy sources (solar and wind) provided an estimated 9% of the city energy supply. Based on the continuation of current trends, by 2030, renewable energy will provide 45%.

**Table 1:** Historical and projected 2030 energy supply (GWh) to Ahmedabad by source (see Supplemental Table S5 for data sources)

Source	2015	2016	2017	2018	2019	2030
APP	1811	1477	1732	1704	1835	2095
SUGEN Power Plant	1613	2353	3263	4434	4269	5263
Bilateral	2754	3024	845	116	45	53
Power exchange	676	180	1129	1060	1340	703
Renewables	128	244	384	729	784	6630
Surplus	106	− 49	62	− 42	68	0
Total energy supply (GWh)	7087	7229	7414	8001	8340	14,744

Table 2 and Fig. 3 display the results of our cool roof inventory analysis. Based on our spatial analysis of land cover in Ahmedabad, we estimate the potential building roof area for modification in 2030 based on an economic growth assumption (World Bank 2020).

**Table 2:** Estimates of potential 2030 roof area in Ahmedabad based on 2018 roof area coverage across all zone types and an economic growth assumption (World Bank 2020), for three total cool roof coverage scenarios (business-as-usual 5%, 12.5%, and 20%)

	2018			2018–2030	2030			
Sector	Plot area (km^2^)	Ratio of plot area to roof area	Total roof area (km^2^)	Annual growth rate in roof area	Total roof area (km^2^)	Total cool roof area (km^2^)		
						BAU 5%	12.5%	20%
Residential	165	36.7%	56	2%	75	3.75	9.38	15.00
Commercial	7	62.9%	4	0.50%	5	0.25	0.63	1.00
Industrial	30	69.7%	20	0.25%	22	1.10	2.75	4.4
Institution	7	16.3%	1	0.15%	1	0.05	0.13	0.20
Total	209		82		103	5.15	12.88	20.60

Our estimates of annual cooling demand for Ahmedabad in 2018 and 2030 are listed in Table 3. Column A indicates the base level of cooling energy demand in 2018 with the existing 5% coverage of cool roofs. Column B displays non-climate effects of population and GDP growth under three cool roof scenarios in 2030 (BAU 5% coverage of available municipal roof area, 12.5%, and 20% coverage). Column C shows the combined climate and non-climate effects in 2030 under the three cool roof coverage scenarios.

**Table 3:** Cooling energy demand in Ahmedabad in 2018 baseline versus 2030 cool roof scenarios (TWh). Column A displays the 2018 baseline, column B indicates the 2030 energy effects of population and GDP growth under three cool roof areal coverage scenarios, and column C the 2030 combined effects of population and GDP growth, plus climate warming, at different levels of cool roof coverage

	Annual cooling energy demand (TWh)						
	2018	2030					
	(A) Baseline	(B) Non-climate effects (GDP and population growth)			(C) Climate and non-climate effects		
Cool roof coverage	BAU 5%	BAU 5%	12.5%	20%	BAU 5%	12.5%	20%
Space cooling	0.87	2.53	2.46	2.40	2.63	2.57	2.50
Refrigeration	0.45	0.99	0.97	0.94	1.03	1.01	0.98
Cold chain	0.03	0.07	0.07	0.07	0.08	0.07	0.07
Industrial process cooling	0.11	0.46	0.44	0.43	0.48	0.46	0.45
Total	1.46	4.05	3.94	3.84	4.22	4.12	4.01

Without considering climate warming, 2030 total cooling energy demand is expected to increase by a factor of 2.77 (extra 2.59 TWh) from 2018 in the BAU cool roofs case (to 4.05 compared to 1.46 TWh, columns A and B), while strong ambition to expand cool roofs to 12.5–20% coverage would reduce that growth in cooling demand by 0.11–0.21 TWh (column B). When climate effects are also considered (column C) and compared to non-climate effects, 2030 total cooling energy demand increases by 0.17 TWh in 2030 in the BAU cool roof case (to 4.22 compared to 4.05 TWh, columns B and C), and strong ambition to expand cool roofs to 12.5–20% coverage would reduce that growth in cooling demand by 0.10–0.21 TWh (column C). This finding suggests that ambitious cool roof expansion by 2030 can compensate for extra cooling energy demand driven by climate warming.

## Discussion

Our analysis of the effects of population growth, economic expansion, and climate change-driven temperature increases on electricity demand in Ahmedabad indicates a near doubling in total electricity supply (Table 1) and a nearly threefold growth in cooling demand (Table 3) between 2018 and 2030. However, renewable energy sources can meet much of this growing demand. Table 1 shows that additional renewable energy capacity is projected to meet about 45% of 2030 energy needs for the city (6630 of 14,744 GWh), compared to about 9% in 2018 (729 of 8001 GWh).

Climate change-driven temperature increase will add 0.17 TWh in cooling energy demand in 2030 relative to 2018, but the 20% cool roof scenario could cut that increased annual demand by 0.21 TWh relative to the 5% BAU cool roof case (comparing results in Table 3 columns B and C). Therefore, our analysis indicates that robust expansion of cool roofs could offset increases in cooling energy demand driven by climate warming. In this way, cool roof actions can advance both mitigation goals (by keeping indoor building temperatures lower where fossil fuels are used to meet cooling and electricity demand) and simultaneously adaptation goals of building resilience to extreme heat.

Our estimate of 0.21 TWh annual reduction in cooling energy demand achieved by 20% cool roof coverage in 2030 is equivalent to 8% of the annual power generation for the Ahmedabad plant (see Supplemental Table S13). That reduction in energy demand corresponds to 191,000 metric tons of avoided carbon dioxide pollution, emissions equivalent to 21.5 million gallons of gasoline (U.S. Environmental Protection Agency 2015). Beyond cool roofs, a preliminary analysis indicates that implementing improved building energy codes can help the state of Gujarat to save nearly 83 TWh cumulatively in energy demand by 2030.

This analysis provides a proof-of-concept for estimating the energy savings from cool roof implementation at the city level and establishes methods for simulating those effects in other areas facing significantly higher future cooling demand due to climate change. This study establishes a framework for integrating data sets across climate, energy, population and economic growth, and the built environment. The spreadsheet tool (see Supplemental Information) is readily modifiable to adapt to other settings and different combinations of climate warming and cool roof deployment.

Cool roofs are an important low-cost passive cooling strategy for India to confront mounting climate risks from extreme heat. Passive cooling technologies are an important strategy embedded within local heat action plans to better protect public health from heat risks. Studies in Hyderabad and Ahmedabad have established that initial material costs are comparable with traditional roofing materials and can be installed in existing buildings. In India, cool roofs can cost as little as five Rupees (USD 0.07) per sq ft. for reflective white lime paint and up to 100 Rupees (USD 1.33) per sq ft. for China mosaic tiles (Natural Resources Defense Council 2020). Slum communities are susceptible to extreme heat because of a lack of access to cooling. Furthermore, housing in these areas is often constructed from heat-trapping materials such as tin sheets, cement sheets (asbestos), plastic, and tarpaulin without sufficient ventilation (Tran et al. 2013). Beyond cool roof benefits for reducing energy demand, evidence indicates that these strategies can also help to moderate the urban heat island effect (Vellingiri et al. 2020) and can ameliorate air pollution (Zhang et al. 2019). We acknowledge these mechanisms as relevant public health effects, but they fall outside the scope of our analysis. In the future, the change in the amount of area available for cool roof implementation relative to air conditioned floor area may decrease if newer buildings are taller than prior ones, and we have not considered this effect (nor the effect of slum redevelopment schemes) within our modeling.

The combined population- and GDP-based scaling method we used provides an approach that resolves national energy demand projections to local contexts. As per IEA estimates, in 2050, India will account for 30% of global emissions from space cooling (International Energy Agency 2018). Sustainable cooling solutions such as cool roofs are also integral to achieving India’s climate goals. Our research provides strong evidence for advancing cool roof policies as part of India’s 2030 climate targets (increasing non-fossil energy capacity to 500 GW to meet half of its energy needs with renewable energy) (United Nations Framework Convention on Climate Change 2021). By reducing cooling and electricity demand from A/C use, cool roofs can also function to reduce carbon dioxide emissions and hydrofluorocarbons (potent heat-trapping gases used as refrigerants in air conditioners) (Velders et al. 2015), supporting the implementation of the ICAP and India’s climate commitments under the Kigali Amendment to the Montreal Protocol.

This work can also be used to advance the implementation of national and state policies to decarbonize buildings, such as the Energy Conservation Building Code (ECBC), because cool roofs are included in the national ECBC and some state ECBC programs (Ministry of Power Bureau of Energy Efficiency 2021). Similarly, this work, by providing an evidence base of the impact of cool roofs, can inform efforts to implement cool roofs as part of state and national housing policies, such as cool roof application on affordable housing under Pradhan Mantri Awas Yojana, the Prime Minister’s Housing Scheme. This work could be used to implement national Heatwave Preparedness Guidelines, local heat action plans, and local cool roof programs as these policies include cool roofs (National Disaster Management Authority (Government of India) (2017); Ahmedabad Municipal Corporation 2019). This work could also be applied to adaptation-specific efforts such as the newly announced International Coalition for Disaster Resilient Infrastructure (OECD 2021).

A number of limitations are apparent in our investigation. We assumed no change in future energy demand for non-cooling needs, and as a result, our 2030 scenario is not representative of the city’s total energy burden. The publicly available energy sector data that we utilized for our analysis was not resolved in adequate temporal detail, which prevented us from understanding seasonal and sub-seasonal (e.g., quarterly and monthly) relationships between climate warming and cooling energy demand. Similarly, our estimate of climate warming in Ahmedabad utilized an annual temperature metric, despite the fact that changes in cooling demand and associated power sector impacts may be most pronounced over the warmest summer months; meteorological data indicate that warming is not uniform across seasons (Sanjay 2020). We relied on RCP 8.5 to estimate local temperature change between 2018 and 2030; estimation of warming beyond 2030 would benefit from finer-scale temperature estimates downscaled from estimaes corresponding to Shared Socio-Economic Pathways (SSPs), in alignment with recent climate modeling available at a broader scale (IPCC 2021).

Due to a lack of available data, we were unable to quantify the temperature and power sector emission effects of other passive cooling measures (e.g., urban greening) despite preliminary evidence indicating that such adaptation efforts can meaningfully reduce local temperatures and address the urban heat island effect (Li et al. 2014; Qin 2015). We isolated our analysis to cool roof impacts on cooling energy demand due to data constraints and did not explore the energy ramifications of other building modifications that could enhance energy efficiency (including energy conservation building codes) and improve indoor thermal comfort (Ramesh and Khan 2013; Subramanian et al. 2017; Yu et al. 2017; Alghamdi et al. 2022). Furthermore, we did not consider the extent to which installation of solar rooftop photovoltaics may affect the availability of building roof area for cool roof installations (Salamanca et al. 2016; Cavadini and Cook 2021). In terms of spatial specificity, our analysis of power sector emissions was regional in nature, but we did not consider the potential downstream implications of changing energy demand in Gujarat for the energy system in other parts of the country, areas that will also be stressed by climate warming in the coming years. We also did not consider the upstream policy actions, implementation challenges, and behavioral changes that would be required in order to scale up cool roof implementation to the degree modeled in this study. A logical next step to further build on this investigation would be to conduct work at a higher temporal resolution and broader geographic scope to better understand intra-annual changes in cooling demand and the cumulative effects on the country’s energy grid.

Moreover, this work does not estimate energy-related air pollution emissions and their associated harms to human health; a separate analysis led by our team investigates the air quality and human health effects of the landcover adaptation scenario analyzed here (Limaye et al. submitted). We did not consider the direct effect of cool roofs on human health (He et al. 2020), nor their impact on potentially reducing the urban heat island effect. However, we are currently analyzing the city-level air quality and health implications of the energy results described in this study. Our future modeling does not consider post-2030 impacts, although evidence indicates that further warming between now and mid- and late-century may push maximum temperatures to 3.93–4.38 °C above the 1976–2005 average (Sanjay 2020). Our analysis assumed equitable access to building cooling in Ahmedabad to enable the modeling, despite the fact that a significant portion of the local population may not able to afford such technology (Tran et al. 2013). This study does not examine the inequitable distribution of A/C in the city and the ways in which cooling energy demand among people with higher household incomes may trigger a disproportionate share of power sector pollution.

We assumed a single metric to capture the impact of cool roofs on cooling energy demand, although other research indicates that the specific reflective surface deployed is key for ultimate albedo and energy savings effects (Vellingiri et al. 2020). We do not account for evidence indicating that cool roof effectiveness may decrease over time (Gaffin et al. 2012). We do not account for the ongoing needs of maintaining cool roofs into the future, nor do we engage in any economic analysis of the relative costs and monetized benefits of the local mitigation and adaptation strategies included in this work despite evidence indicating cool roofs are a cost-effective adaptation strategy (Natural Resources Defense Council 2019).

In a country where about 6% of households have A/C (Romanello et al. 2021), access to affordable cooling can be a matter of survival for millions of people and not just comfort. Studies in Hyderabad and Ahmedabad have established that initial material costs are comparable with traditional roofing materials and can be installed in existing buildings. Slum communities are one of the groups that are the most susceptible to extreme heat because of the lack of access to cooling and that slum housing is often made of heat-trapping materials such as tin sheets, cement sheets (asbestos), plastic, and tarpaulins, without sufficient ventilation. The ICAP also highlights the implementation of cool roofs as a readily actionable cooling solution, through localized heat action plans, particularly for affordable housing projects for the poor (Ministry of Environment, Forest and Climate Change (Government of India) 2019). For example, the southern state of Telangana included a Draft Cool Roofs Policy in its 2021 Heatwave Action Plan (Government of Telangana 2021).

This work establishes a transparent, user-friendly modeling framework for estimating the effects of expanding city-level cool roof programs. A fuller analysis of the energy savings (and corresponding air quality and public health benefits) of cool roofs could help catalyze more rapid uptake of this promising low-cost, low-tech climate resilience measure in other Indian cities by articulating these local benefits in far greater detail than has previously been available. The analytical methods described in this study integrate publicly available climate, land cover, and energy data to estimate energy savings benefits at the municipal level and set out a transparent framework to conduct analyses in other Indian cities.

## Conclusions

Continuing climate warming, population growth, and economic expansion are expected to trigger increased demand for cooling buildings, especially in low-income countries where the current prevalence of A/C is low. Our analysis indicates that efforts to significantly expand cool roof adaptation can help compensate for increased future cooling energy demand. Actions to shift electricity production away from fossil fuels towards renewable energy sources can help to slow long-term climate change and the growing need for cooling.

## Data Availability

All data underlying our analysis are available in the Supplemental Information: 10.5281/zenodo.6434209.

## Appendix

Access electronic supplementary material at: 10.5281/zenodo.6434209.
